# Physician Care Patterns and Adherence to Postpartum Glucose Testing after Gestational Diabetes Mellitus in Oregon

**DOI:** 10.1371/journal.pone.0047052

**Published:** 2012-10-11

**Authors:** Monica L. Hunsberger, Rebecca J. Donatelle, Karen Lindsay, Kenneth D. Rosenberg

**Affiliations:** 1 University of Gothenburg, Public Health Epidemiology and Community Medicine, Gothenburg, Sweden; 2 Oregon State University, Department of Public Health, Corvallis, Oregon, United States of America; 3 UCD Department of Obstetrics & Gynaecology, National Maternity Hospital, Holles Street, Dublin 2, Ireland; 4 Oregon Public Health Division, Portland, Oregon, United States of America; 5 Department of Public Health and Preventive Medicine, Oregon Health & Science University, Portland, Oregon, United States of America; The University of Adelaide, Australia

## Abstract

**Objective:**

This study examines obstetrician/gynecologists and family medicine physicians' reported care patterns, attitudes and beliefs and predictors of adherence to postpartum testing in women with a history of gestational diabetes mellitus.

**Research Design and Methods:**

In November–December 2005, a mailed survey went to a random, cross-sectional sample of 683 Oregon licensed physicians in obstetrician/gynecologists and family medicine from a population of 2171.

**Results:**

Routine postpartum glucose tolerance testing by both family physicians (19.3%) and obstetrician/gynecologists physicians (35.3%) was reportedly low among the 285 respondents (42% response rate). Factors associated with high adherence to postpartum testing included physician stated priority (OR 4.39, 95% CI: 1.69–7.94) and physician beliefs about norms or typical testing practices (OR 3.66, 95% CI: 1.65–11.69). Specialty, sex of physician, years of practice, location, type of practice, other attitudes and beliefs were not associated with postpartum glucose tolerance testing.

**Conclusions:**

Postpartum glucose tolerance testing following a gestational diabetes mellitus pregnancy was not routinely practiced by responders to this survey. Our findings indicate that physician knowledge, attitudes and beliefs may in part explain suboptimal postpartum testing. Although guidelines for postpartum care are established, some physicians do not prioritize these guidelines in practice and do not believe postpartum testing is the norm among their peers.

## Introduction

Gestational diabetes mellitus (GDM) is an important public health concern as it increases the future risk of developing type 2 diabetes for both mother and child [1]. Defined as impaired glucose tolerance first diagnosed during pregnancy, GDM affects approximately 7% of pregnancies a year in the United States [2]. GDM is the most common metabolic complication of pregnancy and its frequency reflects the frequency of type 2 diabetes the underlying population [3].

A systematic review of GDM patients from 1965–2001 found the cumulative incidence of type 2 diabetes increased markedly in the first 5 years after a GDM pregnancy and appeared to plateau after 10 years [4]. There is evidence that in some populations, women with a history of GDM comprise a substantial proportion of the type 2 diabetes population [5]. In a 2009 meta-analysis, women with previous GDM had 7.5-times the risk of developing type 2 diabetes in the future compared to women with normoglycemic pregnancy [6].

There is evidence that lifestyle modification can prevent or delay the development of type 2 diabetes in high-risk populations [7]–[9]. Improving care to women with a history of GDM could reduce the incidence of type 2 diabetes since women with a history of GDM comprise a high-risk group, postpartum care and continued follow-up care should be a priority. Therefore, the US National Diabetes Education Program encourages obstetrician/gynecologists (Ob/Gyns) and primary care providers to better serve the needs of women with prior gestational diabetes [10]. The puerperium period is frequently referred to as an opportune time for a woman's physician to encourage health habits and provide medical therapy that ultimately may improve her quality of life [11]. Physicians are uniquely situated to detect diabetes, deliver care, and educate women with a history of GDM during the prodromal period of disease.

There are inconsistent recommendations for postpartum testing published by the American Diabetes Association (ADA) [12], the American College of Obstetrics and Gynecologists (ACOG) [13], the American Academy of Family Physicians [14], and the Fifth International Workshop-Conference on Gestational Diabetes Mellitus Panel [15]. The American Diabetes Association recommends testing with either fasting plasma glucose (FPG) or an oral glucose tolerance test (OGTT) to reclassify maternal glycemic status [12]. ACOG recommends testing by OGTT to identify a greater number of women with impaired glucose tolerance [13]. The American Academy of Family Physicians recommends that women have their glucose tested prior to discharge and that “regular testing for type 2 diabetes should be strongly encouraged” thereafter [14]. The opinion of the Fifth International Workshop Panel is that women who do not have GDM in the immediate postpartum period should be screened with OGTT 6 to 12 weeks postpartum [15]. The panel considered FPG to be inadequate at identifying women with impaired glucose tolerance [15]. To date, follow-up care has been found to be inadequate in a number of studies [16]–[20]; including the findings from a recent systematic review which reported only half of women in most populations are screened [21].

This study explores the extent to which physicians report performing impaired glucose tolerance testing in the postpartum period in one US state and explores physician characteristics, knowledge, attitudes and beliefs that may elucidate the observed sub-optimal follow-up care that has been previously reported.

## Methods

This cross-sectional study assessed physician knowledge, attitudes, beliefs and practice patterns regarding the care of women with gestational diabetes. A representative sample of Oregon physicians (MDs) holding active licenses with the Oregon Board of Medical Examiners in Family Medicine (FM) and Obstetrics/Gynecology (Ob/Gyn) received a survey at the end of 2005. The total population of MDs in these two specialties was 2171 (1614 FM and 557 Ob/Gyns). A disproportionate random sample of 750 MDs was selected using Stata Intercooled (Version 9; College Station, Texas) with a larger proportion of Ob/Gyn physicians sampled to adequately represent the specialty that most often care for pregnant women. Three-hundred Ob/Gyn physicians and 450 FM physicians were sent surveys using a three pass method in which first a letter of explanation was sent with the initial survey, second a reminder postcard, and third a new cover letter and another copy of the survey over a three week period. This project was approved by the Institutional Review Board at Oregon State University.

The survey was designed to examine physician care patterns both during pregnancy and in the postpartum period; the current study focuses on only the postpartum period. Physicians responded to statements regarding postpartum care knowledge following GDM on a five-point likert-scale (never, rarely, some of the time, most of the time, always) and additional statements reflecting their attitudes and beliefs about glucose testing in the postpartum period using a four-point likert-scale (strongly disagree, somewhat disagree, somewhat agree, and strongly agree). Physicians also responded to one question about the priority of postpartum glucose testing on a four-point likert-scale (very low priority, somewhat low priority, somewhat high priority, very high priority). Likert-scale questions were further dichotomized for summary and analyses in the following manner: five-point likert-scale items never, rarely, sometimes to “no” while most of the time and always were dichotomized to “yes” the four point likert-scale items were dichotomized to “disagree” for strongly disagree and somewhat disagree while somewhat agree and strongly agree were dichotomized to “agree” and the four point likert-scale question regarding priority was dichotomized in the same manner as high and low. The survey questions were presented in a skip pattern format so that if a physician did not work with pregnant women they could skip to the questions regarding postpartum care. Physicians identified their practice area as rural, urban, or suburban based on their own perceptions rather than defined population parameters. All variables included in the analyses were self-reported including the physicians primary practice area. Multivariate logistic regression was used to examine factors that might explain differences in reported adherence to postpartum glucose testing. All analyses were carried out using Stata 11.2, College Station, Texas 77845 USA.

## Results

A total of 285 of 683 eligible physicians participated in the GDM care survey, representing a response rate of 42%. Forty-five MDs did not participate because of inactive status or relocation. Twenty-two physicians identified their primary practice specialty as “other” and they were excluded from the analysis; of these 4 were licensed in FM and 18 in Ob/Gyn. FM physicians returned 166/383 completed surveys, a response rate of 44%, and Ob/Gyn physicians returned 119/300 completed surveys, a response rate of 40%.

A summary of respondent demographic characteristics is shown in Table 1. FM physicians were more likely to be male and rural than Ob/Gyns. Ob/Gyn physicians were more likely to see pregnant women. Of the 285 respondents, 192 worked with pregnant women in their practice. Of the 93 respondents who did not see women during pregnancy, only ten identified themselves as Ob/Gyns.

**Table 1:** Demographics of study respondents by physician specialty (N = 285).

	Family Medicine	Obstetrics/Gynecology	Total
**# of Respondents**	166 (58.0%)	119 (42.0%)	285
**Sex**			
Male	92 (55.0%)	53 (44.5%)	145
Female	75 (45.0%)	65 (54.6%)	140
**Location**			
Urban	54 (32.5%)	35 (29.4%)	89
Suburban	52 (31.3%)	68 (57.1%)	120
Rural	60 (36.1%)	16 (13.5%)	76
**Years in practice**			
<2	13 (7.8%)	6 (5.0%)	19
2–5	32 (19.3%)	21 (17.7%)	53
>5–10	27 (16.3%)	25 (21.0%)	52
>10–20	54 (32.5%)	38 (31.9%)	92
>20	40 (24.1%)	29 (24.4%)	69
**Pregnant patients**			
Yes	83 (49.7%)	109 (92%)	192
No	83(49.7%)	10 (8%)	93
**Type of Practice**			
HMO	12 (7.2%)	19 (16.0%)	31
Private (solo or two)	37 (22.3%)	24 (20.2%)	61
Private group	48 (28.9%)	55 (46.2%)	103
Public health	6 (3.6%)	1 (0.8%)	7
Community clinic	26 (15.7%)	1 (0.8%)	27
Hospital basedUniversity based	3 (1.8%)	7 (5.9%)	10
	10 (6.0%)	7 (5.9%)	17
Other	24 (14.5%)	5 (4.2%)	29

Table 2 summarizes physician responses regarding the care provided to GDM women dichotomized from a series of likert-scale questions. Differences between specialties are examined using the Chi-square test with associated p-values (table 2). Ob/Gyn physicians were more likely to state that they routinely test for glucose intolerance in the postpartum period and were more likely to report they inform women that they are at increased risk for type 2 diabetes. More FM physicians identified believing that postpartum testing following GDM is a cost-effective primary prevention of type 2 diabetes but the differences by specialty are not statistically significant. When asked about the risk for progressing to type 2 diabetes in the future, both FM and Ob/Gyn physicians (73.5% and 69.8% respectively) identified agreement that women are at high risk for progressing to type 2 diabetes. Physicians were also asked to identify agreement with GDM being a transient metabolic condition. Thirty-six percent of Ob/Gyn physicians agreed that GDM was transient while only 18.7% of FM physicians agreed with this statement. Both family physicians and Ob/Gyns responded similarly regarding testing norms but differences by specialty were found regarding priority in practice.

**Table 2:** Physician knowledge of guidelines by specialty (N = 285).

Survey statements	Family Medicine	Obstetrics/Gynecology	Total	p-value
	166 (58.0%)	119 (42.0%)	285	
*If a woman had GDM, I routinely screen for glucose intolerance at her first postpartum visit* a				p<0.001*
Yes	32 (19.3%)	42 (35.3%)	74	
No	86 (51.8%)	71(59.7%)	157	
Not applicable	48 (28.9%)	6 (5.0%)	54	
*I tell post-GDM women that they are at an increased risk for type 2 diabetes* a				p<0.001*
Yes	32 (21.1%)	42 (36.2%)	74	
No	86 (56.6%)	71 (61.2%)	157	
Not applicable	48 (28.9%)	6 (5.0%)	54	
*Testing post GDM women annually is cost-effective primary prevention of type 2 diabetes* b				p>.05
Agree	103 (62.05%)	57 (47.9%)	160	
Disagree	52 (31.3%)	51 (42.9%)	103	
No response	11 (6.6%)	11 (9.2%)	22	
*Over 50% of women with GDM pregnancies will progress to type 2 diabetes within 10 years* b				p>.05
Agree	122 (73.5%)	83 (69.8%)	205	
Disagree	31 (18.7%)	32 (26.9%)	63	
No response	13 (7.8%)	4 (3.4%)	17	
*I feel that GDM is a transient metabolic condition of pregnancy* b				p<.005*
Agree	31 (18.7%)	43 (36.1%)	74	
Disagree	122 (73.5%)	72 (60.5%)	194	
No response	13 (7.8%)	4 (3.4%)	17	
*Most physicians provide follow-up glucose testing after a GDM pregnancy* b				p>.05
Agree	64 (38.6%)	41 (34.5%)	105	
Disagree	85 (51.2%)	70 (58.8%)	155	
No response	17 (10.2%)	8 (6.7%)	25	
*To what extent is testing post GDM women a priority in your practice* c				p<.05*
Low	96 (57.8%)	71 (59.7%)	167	
High	55(33.13%)	46 (38.7%)	101	
No response	15 (9.0%)	2 (1.7%)	17	

a . 5 point likert-scale dichotomized (never, rarely, sometimes = no and most of the time and always = yes).

b . 4 point likert-scale dichotomized (strongly disagree, somewhat disagree = disagree and somewhat agree and strongly agree = agree).

c . 4 point likert-scale dichotomized (very low priority and somewhat low priority = low and somewhat high priority and very high priority = high).

* significant difference between FM and Ob/Gyn.

Logistic regression results (table 3) indicate that adherence to postpartum glucose testing is explained in part by two variables, priority in practice and practice norms. Physicians that considered postpartum glucose testing to be a priority (OR 4.39, 95% CI: 1.69–7.94) and those that believed testing to be typical or a practice norm (OR 3.66, 95% CI: 1.65–11.69) were more likely to indicate adhering to guidelines to reclassify glycemic status in the postpartum period. Postpartum testing did not differ by physician specialty, sex, years of practice, or type of medical practice.

**Table 3:** Predictors of postpartum glucose testing adherence, multivariate model.

	Odds Ratio	95% CI
Typical of others^a^	3.66	1.69–7.94*
Priority in practice^b^	4.39	1.65–11.69*
Specialty of physician^c^	2.02	0.88–4.62
Sex of physican d	1.34	0.59–3.09
Urban practice location^e^	0.58	0.23–1.48
Rural practice location^e^	1.49	0.54–4.13
Years Practice^f^	0.98	0.94–1.03
HMO type practice^g^	2.81	0.85–9.27
Solo type practice^g^	1.20	0.38–3.83
Public type practice^g^	1.49	0.52–4.24
Community type practice^g^	1.04	0.23–4.66
Cost-effective^h^	1.42	0.54–3.71
Represent high risk group for future type 2 diabetes^i^	0.35	0.04–2.82
Progression to type 2 diabetes is likely j	0.58	0.24–1.39
Transient metabolic condition^k^	0.98	0.41–2.35

a respondent agreed that most physicians provide follow-up glucose testing after a GDM pregnancy; referent: No.

b respondent said that screening post GDM women was a priority in their practice; referent: Not a priority.

c Referent: family medicine.

d Referent: male.

e categorical dummy variable - suburban practice location as the referent.

f continuous variable.

g categorical dummy variable - private group practice is the referent.

h respondent agreed that testing post-GDM women annually is cost-effective; referent: No.

i respondent agreed that women with GDM were at high risk for future type 2 diabetes; referent: disagreed.

j respondent agreed that progressing to type to is likely; referent: No.

k respondent agreed that GDM is a transient metabolic condition; referent: No.

* significant predictor of postpartum glucose testing.

## Discussion

We found postpartum testing for women with a history of GDM was not routinely practiced in this statewide sample. Although guidelines for postpartum care are established, many physicians do not prioritize these guidelines in practice and do not believe postpartum testing is the norm among their peers. Our findings indicate that physician beliefs and attitudes partly explain suboptimal postpartum testing while other physician characteristics do not seem to influence practice patterns.

Although our study relies solely on self-reported physician practice patterns, suboptimal postpartum glucose testing was reported. Other studies examining postpartum testing have also found suboptimal postpartum glucose testing. A systematic review reported only half of women in most populations are screened [21] Stasenko *et al.* reported a 33.7% follow-up in a US academic center cohort [22]. In a recent study that included approximately 800,000 women, only 19% of women with GDM received postpartum diabetes testing within a 6-month period [17]. Our findings are similar to those of a survey conducted with family medicine physicians in Texas in which 33% of respondents reported providing follow-up care to a patient with GDM [22], and those of Blatt *et al.* [17] which relied on a review of women who used the services of Quest Diagnostics. A 2009 publication, reported an increase in postpartum screening between 1995 and 2006 but screening was still suboptimal at 53% in a large cohort from six translation research centers [20]. One surprising finding of our study is that postpartum glucose testing was suboptimal even though approximately 70% of physicians reported always warning women about their increased risk for type 2 diabetes. Potential explanations may include physicians do not believe that they can delay the onset of type 2 diabetes, they may view another health practitioner as responsible for the follow-up, or the patients fail to return but physicians do understand that the patients are at increased risk.

The practice type to which a woman is referred for postpartum follow-up care may influence the likelihood of her receiving a glucose tolerance test. Russell et al. reported that women attending follow-up visits at a hospital-based clinic, as opposed to a hospital-affiliated community clinic, were twice as likely to complete the recommended postpartum glucose testing [16]. However, our findings did not support practice type as a significant predictor of reported postpartum glucose testing.

A lack of consensus regarding when to test, which test to perform, and who is responsible may create a barrier to postpartum testing. Although the American Diabetes Association guidelines [12] recommend that glucose tolerance testing be performed at a minimum of three year intervals, more recent guidelines suggest annual testing, since women with a history of GDM are most at risk of type 2 diabetes development in the first 10 years postpartum [23]. One study conducted in England found primary and secondary care doctors disagreed about the tests and responsibilities for follow-up [24]. Additionally, a recent study examined the usefulness of Hemoglobin A_1C_, alone or combined with a fasting glucose test, compared to an oral glucose tolerance test for use in the postpartum period as a means of reclassify women with a history of GDM [25]. This may suggest that there is dissatisfaction with existing recommendations. However, the authors did conclude that their results indicate the hemoglobin A_1C_ test alone or in combination with fasting glucose was not sensitive or specific enough for diagnosis [25]. Further, it is possible that physicians' perceive factors such as affordability of care, insufficient appointment time, inadequate reimbursement, lack of resources and lack of support personnel as the barrier to more frequent glucose tolerance testing. These factors have been identified as barriers to the provision of quality diabetes care among community health centers [26], [27].

The influence of respected peers may also be a barrier. Physicians may be aware of, understand and agree with clinical practice guidelines, but their decision to adopt and act on new information is often influenced by the perceived opinions of their respected peers [28].The social influence theory describes how factors such as custom and habit, assumptions and beliefs of peers, prevailing practices and social norms define and shape the interpretation of information obtained through educational means [29]. The failure of physicians to adopt practice guidelines for the postpartum care of GDM women may, in part, be explained by this theory. When we assessed the beliefs and attitudes of physicians, only 7% of respondents reported to ‘strongly agree’ that ‘most physicians provide follow-up glucose testing’. Therefore, as per the social influence theory, physicians who believe that the majority of their peers are not implementing postpartum practice guidelines may consider follow-up practices to be against the social norm and hence, adoption of testing behavior is low.

Additionally, postpartum women may have their own set of barriers. Bennett et al. explored barriers and facilitators in a qualitative study with 22 postpartum women [30]. Reported barriers included having a baby with increased health needs, personal and family adjustment, the stress of a new baby, demands of a baby's schedule, experiences with medical care and services, dissatisfaction with care, and fear of receiving bad news at an appointment. Canadian patients reported time pressure as the most common reason for a lack of follow-up [31]. Facilitators included the availability of child care, wanting a check-up to be cleared for return to work, a connection to clinical and office staff, appointment timing, specific concerns or questions being addressed [30]. Additionally, 85% of patients surveyed in a Canadian sample reported they perceived reminders as helpful [31]. A qualitative assessment of perceived barriers among women in Atlanta, Georgia found most women were unaware of their risk for developing type 2 diabetes later [32]. This finding could indicate that their future risk was not properly communicated or that these women failed to understand. Further, a review of health beliefs among women with previous gestational diabetes concluded that women had low risk perceptions for future type 2 diabetes mellitus [33]. This suggests either a lack of communication between health providers and patients or a lack of patient understanding. A study of beliefs about health and illness in a Swedish population reported that women were informed by healthcare professionals about gestational diabetes being transient [34]. Healthcare professionals may be inadvertently creating patient barriers by providing misinformation. We did not explore facilitators and barriers from the patient perspective but acknowledge that patient barriers partially explain suboptimal follow-up.

A number of recent studies have explored factors that might increase screening [31], [35]–[41]. Several of these studies have focused on reminders in different formats [31], [35]–[38]. A Canadian study found postpartum postal reminders enhanced screening in the postpartum period [31]. Lawrence *et al.* reported that automated orders for follow-up testing in parallel with notifications to physicians and electronically generated reminders to patients by telephone and email may increase follow-up [36]. Another study found a telephonic nurse management program was associated with increased postpartum testing [37]. Further, the use of a letter or phone call increased oral glucose tolerance testing from 14% to 28% within six months postpartum in another Canadian study [38]. A US study reported educational counseling delivered by a nurse educator in the antepartum period (37–38 weeks) increased postpartum testing [35]. In addition to reminders and counseling, improved communication between primary care and secondary care are reportedly important [41]. Morrison et al report that a team approach to managing care may reinforce the need for postnatal diabetes testing [39] and Lapolla *et al.* reported that improved communication between patients and health care provider team are recommended [40].

Prevention measures are critical; women of reproductive age are currently experiencing increases in the prevalence of chronic diseases such as type 2 diabetes [42]. Future research should explore mechanisms for removing physician barriers to providing comprehensive postpartum care. For instance, a lack of awareness or knowledge about a woman's increased diabetes risk for type 2 diabetes after a GDM-affected pregnancy may contribute to suboptimal postpartum testing. We found that 26% of physicians surveyed believed that GDM is a transient metabolic condition of pregnancy and are, therefore, unlikely to consider postpartum glucose testing a priority. This is consistent with an earlier survey study in which family practice physicians reported inadequate knowledge concerning postpartum glucose testing and future risk for type 2 diabetes following a pregnancy complicated by GDM [22]. Another barrier may be confusion over postpartum practice guidelines. A survey of Ohio practitioners reported use of 2-hr 75 gram OGTT, fasting blood sugar, other, home self-monitoring, hemoglobin A1C, and random glucose among those that report offering follow-up care [43]. The American Diabetes Association [12], the American College of Obstetricians and Gynecologists [13], the Fifth International Congress Workshop for Gestational Diabetes [15], the International Diabetes Federation [23], and the Canadian Diabetes Association [44] all recommend follow-up glucose testing at approximately six weeks postpartum, although the suggested method of testing, e.g., OGTT versus FPG, and the required frequency of follow-up testing after the initial postpartum visit varies. This may be causing confusion that leads to inactivity. The fragmented medical system could also pose a barrier as reported in a Canadian study [31]. In the US, a woman could be diagnosed with GDM by an obstetrician or family medicine doctor but then may be referred to an endocrinologist for care during the pregnancy and then referred back to the primary care provider after delivery. We suffer from the same fragmentation.

The main strength of this study is its investigation of practice patterns, including knowledge, attitudes and beliefs, among a random sample of physicians who are responsible for providing postpartum care to women with a history of GDM. Our study supports earlier evidence that poor clinical awareness of gestational diabetes, methods of testing and local guidelines affect the standard and consistency of care provided [45]. However, our findings demonstrate that awareness of standards is insufficient; the importance of physician prioritization and practice norms in medical practice may be as important as other factors that promote or inhibit follow-up testing.

This study has the following limitations. First, the response rate is low but typical for physician surveys. Second, the study relies on self-reported practice patterns and, therefore, all questions may not have been answered truthfully, or responses could have been influenced by social desirability bias. Third, all physicians surveyed were based in a single US state and, therefore, the results may not be reflective of physician practice patterns throughout the US. Forth, the survey content may have limited the results as only physicians were assessed and therefore, we could not consider the influence of patient-related factors, organizational, structural, philosophical and financial barriers that may have affected postpartum glucose tolerance testing. Finally, data was collected a number of years ago, but despite this limitation, our findings seem to be supported by more recent studies which indicate testing is suboptimal, health practitioners continue to inform their patients that GDM is transient in some cases, and that postpartum screening is still not a universal norm.

Postpartum glucose testing is low and this may be influenced by priorities in practice and practice norms. Clear, non-conflicting postpartum practice guidelines should be agreed upon and adopted to aid in removing potential barriers. Clear messaging to physicians would likely improve adherence to postpartum follow-up testing. Physicians may need help in understanding their role in the broader, public health system that aims to modify health risks and promote women's health.
